# Case Report: Neuroretinitis following combination checkpoint inhibition therapy with nivolumab and ipilimumab for stage IIIC melanoma

**DOI:** 10.3389/fonc.2026.1872343

**Published:** 2026-09-03

**Authors:** Nuha S. Arefin, Jared Moon, Aaron B. Roller, Moe H. Aung

**Affiliations:** 1 Department of Ophthalmology, Dell Seton Medical Center at The University of Texas, Austin, TX, United States; 2 Austin Retina Associates, Austin, TX, United States

**Keywords:** immune checkpoint inhibitor, immune-related adverse event, ipilimumab, neuroretinitis, nivolumab

## Abstract

**Purpose:**

Neuroretinitis is a very rare but recognized immune-related adverse event (irAE) of immune checkpoint inhibitor (ICI) therapy. We present a case of likely ICI-associated neuroretinitis following combination nivolumab and ipilimumab treatment for stage IIIC cutaneous melanoma.

**Observations:**

Our patient is a 48-year-old woman with stage IIIC melanoma who developed acute left eye vision loss approximately three months after initiating nivolumab and ipilimumab, near the end of a corticosteroid taper for a concurrent systemic irAE. Examination showed best-corrected visual acuity of 20/400, severely reduced color vision (2/14 Ishihara plates), and a relative afferent pupillary defect in the left eye. Initial fundus exam of that eye revealed optic disc edema with nasal hemorrhages as well as peripapillary macular edema. There was a central scotoma with nasal steps on the Humphrey visual field (mean deviation -13.24 dB). Fluorescein angiography demonstrated disc leakage with inferior arcuate hypoperfusion. Optical coherence tomography (OCT) showed subsequent intraretinal exudates in the nasal macula bilaterally, worse in the left eye. Infectious workup and neuro-imaging were unremarkable. The patient was treated with intravenous methylprednisolone (IVMP) pulse and intravenous immunoglobulin (IVIG) at initial hospitalization, followed by a slow oral prednisone taper along with monthly IVIG outpatient. Her visual acuity in the left eye improved to 20/20 at 6-month follow-up visit.

**Conclusions and importance:**

ICI-associated neuroretinitis is rare but vision-threatening and may emerge or worsen during systemic corticosteroid tapering for concurrent irAEs. Prompt recognition, exclusion of infectious mimics, and multidisciplinary management can yield meaningful visual recovery. Comprehensive neuro-ophthalmic assessment for ICI-associated neuro-ophthalmic complications including evaluation of color vision and pupillary function, documentation of fundus appearance, and additional ancillary testing with visual field and OCT analysis is essential.

## 1 Introduction

Neuroretinitis is an inflammatory disorder characterized by optic disc edema with inflammation of the peripapillary retina and subsequent formation of lipid exudates that deposit along Henle’s fiber layer in a classic “macular star” pattern as proteinaceous fluid tracks from the swollen disc toward the fovea. Although a complete macular star is considered the classic manifestation, many cases do not form a full macular star but rather peripapillary retinal exudates in the pattern of the regressed macular swelling. The condition carries a broad differential diagnosis but it is critical to often assess for infectious etiologies first with casual pathogens such as *Bartonella henselae, Treponema pallidum, Borrelia burgdorferi*, and *Toxoplasma gondii*. However, systemic inflammatory conditions and toxic etiologies, such as immune checkpoint inhibitor (ICI) therapy, must also be considered (1).

Ocular immune-related adverse events (irAEs) remain uncommon overall with an incidence of 1%, with non-infectious uveitis representing one of the more frequently reported ocular toxicities with incidences ranging anywhere from 0.3% to 6% (2). Separately, ICI-associated neuro-ophthalmic irAEs occurred in only 0.46% (109 out of 23,696) of patients taking ICIs with optic neuritis accounting for 12.8% (14 out of 109) and neuroretinitis only accounting for 0.9% (1 out of 109) of all neuro-ophthalmic irAEs, with a median time to symptom onset of roughly 14.5 weeks after ICI initiation (2). The pathogenesis is thought to involve T-cell cross-reactivity with optic nerve antigens following checkpoint-mediated immune dysregulation (3). Among these optic nerve complications, neuroretinitis is exceptionally rare. To our knowledge, only one prior case of ICI-associated neuroretinitis has been reported in the literature, specifically bilateral neuroretinitis with anterior uveitis following single ipilimumab monotherapy, as described by Hahn and Pepple in 2016 (4). Moreover, combination therapy, such as use of nivolumab (a programmed death 1 [PD-1] checkpoint inhibitor) and ipilimumab (a cytotoxic T-lymphocyte-associated antigen 4 [CTLA-4] checkpoint inhibitor) together, has been found to drive more severe treatment-requiring immune toxicity in up to 55% of patients, a substantially higher rate than either agent alone, reflecting the additive immunologic burden of dual checkpoint inhibition (5, 6).

What makes our case clinically distinctive is threefold: (1) it represents the first reported case of neuroretinitis attributable to combination treatment with nivolumab and ipilimumab; (2) visual symptoms emerged not during active ICI infusion, but at the conclusion of a corticosteroid taper for a concurrent multisystem irAE - suggestive of steroid-withdrawal unmasking pattern with direct management implications; and (3) the patient required escalating immunosuppression with intravenous immunoglobulin (IVIG) after failing corticosteroid monotherapy. Furthermore, we present this case to emphasize the potential need for active ophthalmologic surveillance during corticosteroid tapering in ICI-treated patients.

## 2 Case description

Oral informed consent was obtained from the patient for publication of this de-identified case report in accordance with institutional guidelines.

Our patient is a 48-year-old woman with stage IIIC cutaneous melanoma (metastatic to left upper extremity lymph nodes) who received two cycles of combination therapy of nivolumab (Opdivo) and ipilimumab (Yervoy) approximately one month apart. She subsequently developed significant systemic irAE characterized by hepatotoxicity, altered mental status with hyponatremia, and headaches, for which she was treated with a six-week course of oral prednisone. Toward the end of this taper, she developed sudden blurred vision in the left eye, pain with extraocular movements, and worsening headaches.

She was evaluated by a retina specialist approximately one week after symptom onset, in which optic disc edema with accompanying peripapillary macular inflammation (seen as intraretinal hypo-reflective intraretinal fluid on optical coherence tomography) was identified in the left eye. Fluorescein angiography (FA) demonstrated disc leakage and inferior arcuate hypoperfusion, with milder findings in the right eye. Neuroretinitis versus optic neuritis in the setting of ICI use was suspected, and she was recommended to present to the emergency department for further urgent work-up and management. Magnetic resonance imaging (MRI) of the orbits showed no optic nerve enhancement, and magnetic resonance venogram (MRV) of the head incidentally revealed moderate to severe focal stenosis of the right mid transverse sinus. Lumbar puncture showed elevated cerebrospinal fluid (CSF) protein and immunoglobulin levels, but with normal opening pressure (22 cm H2O, normal: 10–25 cm H2O) and no pleocytosis. Aquaporin-4 antibody (AQP-4: marker for neuromyelitis optica (NMO)) was not detected in the CSF. She was hospitalized and treated with four days of intravenous methylprednisolone (IVMP) pulse and intravenous immunoglobulin (IVIG). She was then discharged on oral prednisone taper.

She presented to our neuro-ophthalmology clinic approximately four weeks after the acute presentation on oral prednisone 40 mg daily. She reported persistent aching pain behind the left eye, was requiring hydromorphone every other day for headache management, and noted no subjective improvement in vision. She reported side effects of fatigue, insomnia, weight gain, and mood change on her current prednisone dose. The patient denied any family history of neurologic or ocular disease, and no report of early-onset blindness in the family. She did not have any recent exposure to ticks, cats, or travel to wooded areas, and did not have any significant substance use history besides infrequent social drinking.

## 3 Physical examination and clinical findings

On examination at our clinic, best-corrected visual acuity (BCVA) was 20/25 in the right eye (pinhole 20/20, OD) and 20/100 in the left eye (pinhole 20/80, improved from 20/400 at onset, OS). Color vision was full in the right eye (14/14 Ishihara plates) but severely reduced in the left eye (2/14 plates). Confrontation visual fields revealed a superonasal quadrantanopia in the left eye. A relative afferent pupillary defect (RAPD) was present in the left eye. Intraocular pressures were 12 mmHg OD and 14 mmHg OS. Slit-lamp examination showed 1+ nuclear lens changes bilaterally with an otherwise unremarkable anterior segment. Extraocular movements were full, but the patient reported subjective pain at extreme gazes.

**Figure 1:**
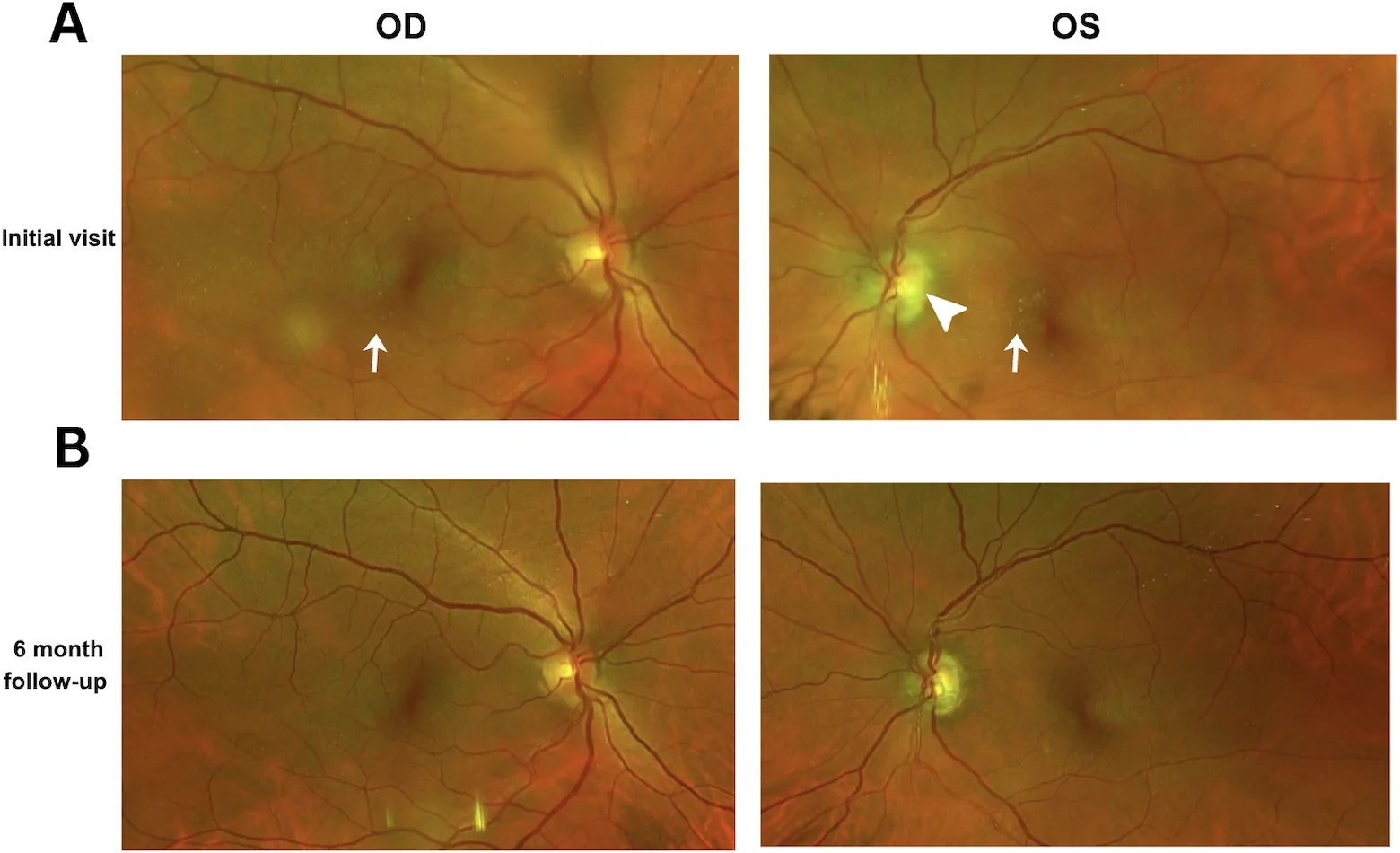
Fundus images of our patient’s right (OD) and left (OS) eye at initial and 6-month follow-up visits. **(A)** Fundus photographs at the initial visit to our institution. Right eye demonstrated a normal-appearing optic disc with scattered exudates in the inferior macula (white arrow). Left eye showed optic disc swelling (white arrowhead) with exudates in the nasal macula (white arrow). **(B)** Fundus photographs at 6-month follow-up. Right eye appeared normal. Left eye demonstrated resolved disc edema with mild temporal disc pallor as well as resolution of intraretinal hemorrhages and exudates.

Dilated funduscopic examination of the right eye showed a cup-to-disc ratio of 0.3, pink and sharp disc margins, but scattered exudates inferior to the macula. On the other hand, left eye fundus showed a swollen optic disc with mild pallor, nasal intraretinal hemorrhages adjacent to the optic nerve head, and exudates nasal to the macula (**Figure 1A**).

**Figure 2:**
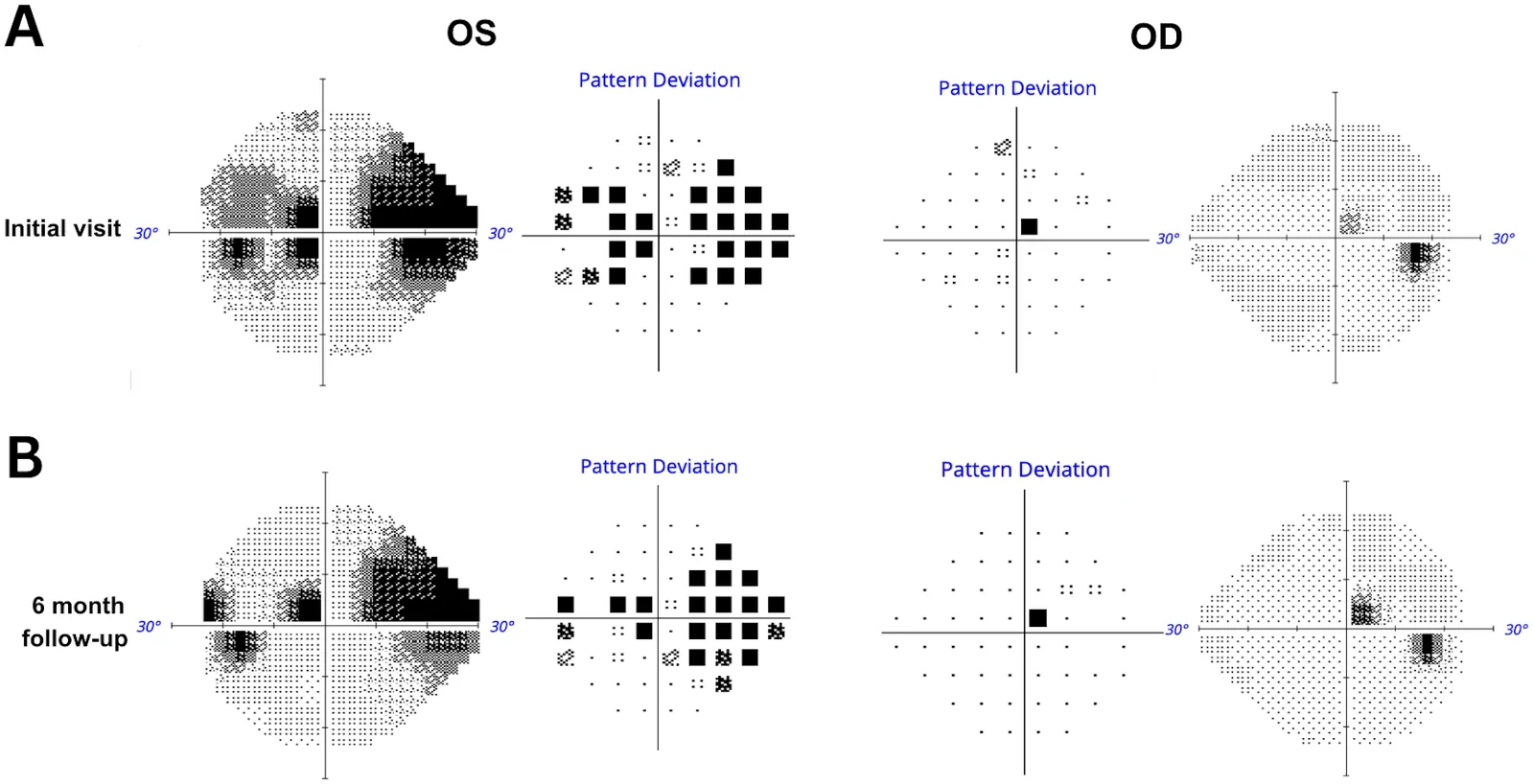
Humphrey visual field (HVF) 24–2 results at initial and 6-month follow-up visits. **(A)** At the initial visit to our institution: left eye (OS) exhibited a cecocentral scotoma with nasal steps and a mean deviation of −13.24 dB, consistent with significant afferent visual pathway dysfunction, while right eye (OD) demonstrated mild supracentral scotoma with a mean deviation of −0.88 dB. **(B)** At 6-month follow-up: left eye showed persistent cecocentral scotoma with nasal steps and mean deviation of −10.24 dB, representing partial improvement. Right eye is stable with mean deviation of +0.24 dB.

**Figure 3:**
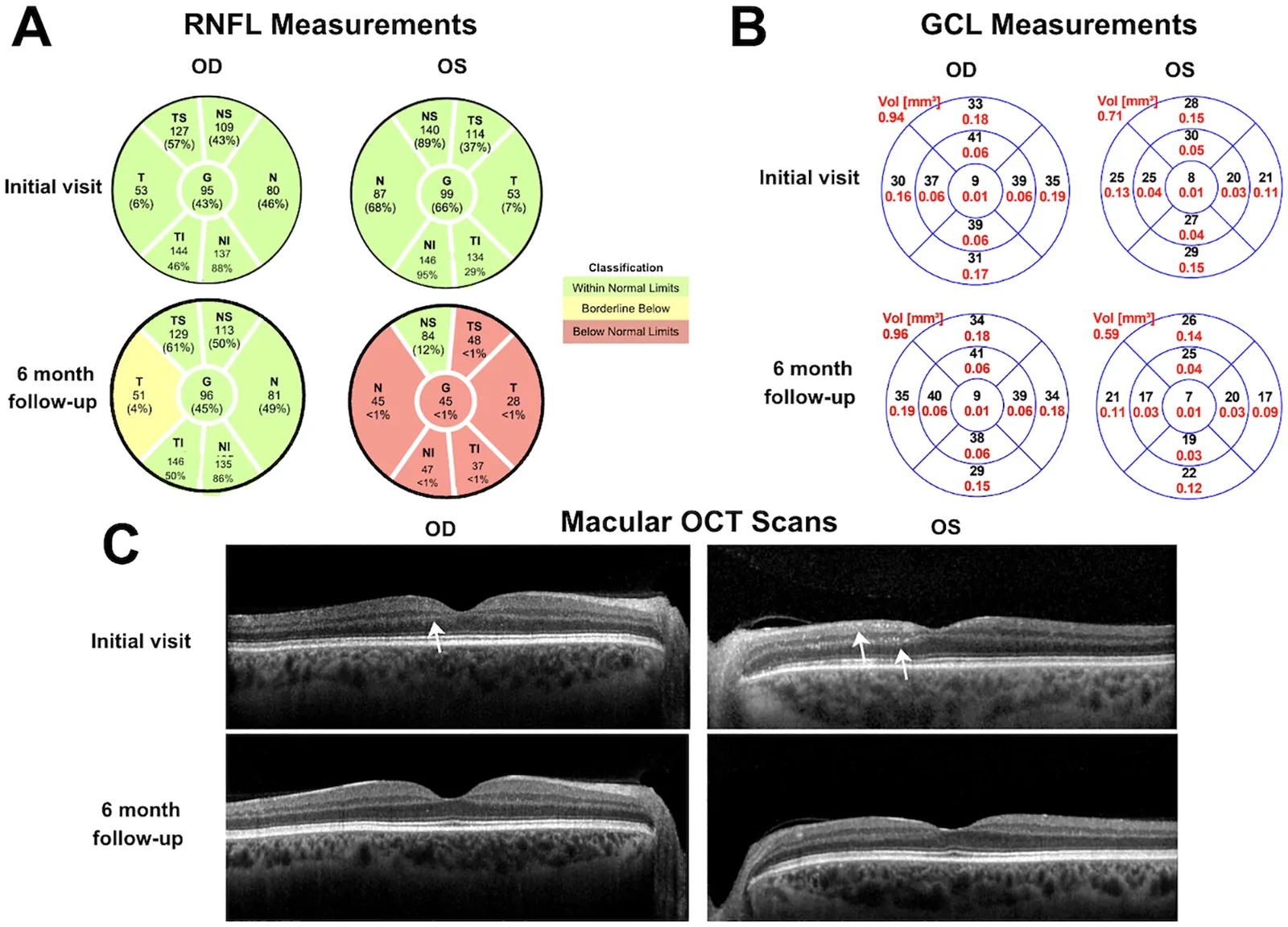
Optical coherence tomography (OCT) analyses at the initial visit and 6-month follow-up. **(A)** Retinal nerve fiber layer (RNFL) sector thickness maps for the right eye (OD) and left eye (OS). At the initial visit, global RNFL was within normal limits bilaterally (OD 95 µm, OS 99 µm) with subtle temporal sector thinning in the left eye. At 6-month follow-up, the right eye remained stable (OD 96 µm), while the left eye showed marked global RNFL thinning (OS 45 µm), consistent with optic atrophy. **(B)** Macular ganglion cell layer (GCL) volume maps for OD and OS. At the initial visit, macular GCL volume was mildly reduced in the left eye (OS 0.71 mm³) when compared to the right eye (OD 0.94 mm³). At 6-month follow-up, GCL volume declined further in the left eye (OS 0.59 mm³), reflecting progressive ganglion cell loss, while the right eye remained stable (OD 0.96 mm³). **(C)** Macular OCT B-scans of OD and OS. At the initial visit, significant intraretinal exudates are visible in the outer plexiform layer (OPL), inner nuclear layer (INL), and inner plexiform layer (IPL) nasal to the macula in the left eye (white arrow), more so than in the right eye. At 6-month follow-up, intraretinal exudates have nearly completely resolved in both eyes.

Additional testing with Humphrey visual field (HVF) 24–2 revealed a cecocentral scotoma with large nasal steps in the left eye (mean deviation −13.24 dB) and a mild supracentral scotoma in the right eye (mean deviation −0.88 dB) (**Figure 2A**). Optical coherence tomography (OCT) analysis at the initial visit showed global retinal nerve fiber layer (RNFL) thickness within normal limits in both eyes (OD 95 µm, OS 99 µm), though with subtle temporal sector thinning bilaterally, suggestive of early disruption of the papillomacular bundles (**Figure 3A**). Macular ganglion cell layer (GCL) volume was already reduced in the left eye (0.71 mm³) when compared to the right eye (0.94 mm³) (**Figure 3B**). Macular OCT scans demonstrated intraretinal exudates bilaterally: more prominent exudates in the outer plexiform layer (OPL), inner nuclear layer (INL), and inner plexiform layer (IPL) nasal to the macula in the left eye when compared to the right eye (**Figure 3C**). Given the exam findings and work-up thus far, she was diagnosed with ICI-associated neuroretinitis affecting both eyes, with the left eye more severely affected than the right eye. Although it is possible that her presentation represents inflammatory optic neuritis with secondary retinal exudation rather than classic neuroretinitis, we favored neuroretinitis because of the combination of optic disc edema with subsequent retinal lipid exudation as well as no evidence of optic nerve enhancement detected on MRI imaging.

At 6-month follow-up after ongoing therapies discussed below, her BCVA recovered to 20/20 in both eyes. Despite remarkable central acuity improvement, significant afferent dysfunction persisted in the left eye: color vision remained reduced (4/14 Ishihara plates) and a 2+ RAPD was still present. Reassuringly, dilated funduscopic examination of the left eye now showed resolved disc edema, mild temporal disc pallor, and complete resolution of the previously noted intraretinal hemorrhages and exudates (**Figure 1B**). Repeat HVF 24–2 testing demonstrated partial improvement in the left eye (mean deviation −10.24 dB), though a cecocentral scotoma with nasal steps persisted; the right eye was stable with questionable mild central scotoma (mean deviation +0.24 dB) (**Figure 2B**). OCT RNFL revealed marked thinning in the left eye: global RNFL measurements reduced from 99 to 45 µm, consistent with interval optic atrophy, while the right eye remained stable at 96 µm (**Figure 3A**). Macular GCL volume in the left eye declined from 0.71 to 0.59 mm³, reflecting progressive ganglion cell loss, while the right eye was stable at 0.96 mm³ (**Figure 3B**). Macular OCT scans demonstrated near-complete resolution of intraretinal exudates in both eyes compared to the initial visit (**Figure 3C**).

## 4 Diagnostic assessment, therapeutic intervention, and outcomes

For our patient, serum testing for infectious etiologies including *Bartonella henselae, Treponema pallidum, Toxoplasma gondii, Borrelia burgdorferi*, and *Mycobacterium tuberculosis* was negative at multiple timepoints. CSF showed elevated protein (76 mg/dL), albumin (52 mg/dL), and IgG (7.4 mg/dL) with normal opening pressure, no pleocytosis, and negative NMO/AQP4 antibody testing. MRI showed no optic nerve enhancement. Taken together, these findings support a clinical diagnosis of neuroretinitis secondary to ICI therapy. The diagnosis was established by clinical and exam findings, a negative infectious workup, absence of optic nerve enhancement on MRI, and CSF findings not consistent with infectious or demyelinating etiologies. Additionally, paraneoplastic syndromes such as cancer-associated retinopathy (anti-recoverin) and anti-collapsin response mediator protein-5 (CRMP-5) associated optic neuropathy were tested for and found to be negative. These diagnoses were also considered less likely because her ocular symptoms and likely neuroretinitis developed after her melanoma was already treated with combination immune checkpoint inhibitor therapy and while she was having multisystem immune-related adverse events.

During the acute inpatient admission, the patient received four days of IVMP pulse and IVIG and then was discharged on oral prednisone 70 mg daily with a 10 mg weekly taper. At our institution’s initial visit, given incomplete recovery, the steroid taper was slowed to 10 mg every two weeks. Gabapentin 300 mg daily was added for neuropathic pain. Referral was placed to neuro-oncology for outpatient IVIG as adjunctive therapy, which was initiated as monthly infusions and continued through the 6-month follow-up visit. Further ICI cycles were not planned or indicated, as the patient had completed the planned course of nivolumab-ipilimumab immunotherapy for her melanoma prior to presentation to our neuro-ophthalmology clinic. Moreover, rechallenge was not pursued due to the severity of her irAEs. Return precautions were discussed, with plasma exchange (PLEX) identified as an escalation strategy should vision deteriorate – PLEX was ultimately not required.

Visual acuity in the left eye improved from a nadir of 20/400 to 20/100 (pinhole 20/80) at our institution’s initial visit, and further to 20/20 at 6-month follow-up with ongoing monthly IVIG. HVF for her left eye improved from a nadir mean deviation of −18.72 dB (peripheral constriction) to −13.24 dB (central scotoma with nasal steps) at our institution’s visits. Serial FA confirmed progressive improvement in disc leakage, and fundus examination demonstrated resolution of disc edema, intraretinal hemorrhages, and exudates, with development of mild temporal disc pallor. The right eye vision remained well-preserved throughout (BCVA 20/20, color 14/14, MD + 0.24 dB). Despite the acuity recovery, significant structural and functional deficits persisted at 6-month follow-up (see above). This dissociation between Snellen acuity recovery and ongoing structural loss highlights the importance of multimodal evaluation at follow-up visits beyond chart acuity in ICI-associated optic nerve disease. Neuro-oncology continued monthly IVIG, and rituximab was under consideration for persistent CSF pleocytosis.

## 5 Episode of care timeline

**Table 1** summarizes the clinical course from ICI initiation through 6-month follow-up at our institution.

**Table 1:** Chronological episode of care timeline.

Timepoint	Event/intervention	Clinical/diagnostic findings	Treatment	VA (left eye)
4 months before vision loss	Cycle 1: Nivolumab + Ipilimumab	Stage IIIC melanoma diagnosed; first ICI cycle initiated	Nivolumab + Ipilimumab	Not recorded
3 months before vision loss	Cycle 2: Nivolumab + Ipilimumab	Second ICI cycle administered	Nivolumab + Ipilimumab	Not recorded
1–2 months before vision loss	Systemic irAE; oral steroid course	Hepatotoxicity, AMS with hyponatremia, headaches	6-week oral prednisone taper (completed Month 4)	Not recorded
Time 0 – Symptom Onset	Visual symptoms onset (end of steroid taper)	Left eye blurry vision, pain with EOMs, worsening headaches	Awaiting evaluation	20/400 (nadir)
Week 2	Evaluation by retina specialist	Optic disc edema in OS; FA with disc leakage, inferior arcuate hypoperfusion in both eyes (OS > OD); neuroretinitis vs. optic neuritis suspected	Transferred to the ED	20/400
Week 2, (Day 2)	ED workup and inpatient admission	MRI brain and orbits: no optic nerve enhancement; MRV: moderate-severe focal stenosis right mid transverse sinus (incidental); LP: OP 22 cm H_2_O (normal 20–25 cm H2O), protein 76 (high), albumin 52, IgG 7.4 mg/dL (high), AQP4-IgG negative; serum CRP 4.1 (high)	Four-day IVMP pulse + IVIG; discharged on prednisone 70 mg daily with 10 mg weekly taper	20/400 → 20/200
Month 1	Follow-up evaluation by retinal specialist	Serial FA: interval improvement in disc leakage on both studies; HVF 24-2: OD MD -1.66 (nonspecific), OS MD -18.72 (peripheral constriction)	Ongoing prednisone taper; PPI + Bactrim (*Pneumocystis jirovecii* pneumonia prophylaxis)	20/200 → 20/80
Month 2	Presentation to neuro-ophthalmology	Color vision OD 14/14, OS 2/14; RAPD OS 3+; CVF: superonasal quadrantanopia OS; fundus: mild disc pallor, blurred margins 360°, nasal hemorrhages and exudates OS; scattered exudates inferior to macula OD; HVF 24-2: OD MD −0.88 (non-specific), OS MD −13.24 (central scotoma + nasal steps); RNFL OCT: OD 95 µm, OS 99 µm; GCL OCT: OD vol 0.94 mm³ (mild exudates), OS vol 0.71 mm³ (significant exudates OPL/INL/IPL); infectious panel negative (Bartonella, RPR, toxoplasma, Lyme, QFT-Gold Plus); negative anti-recoverin and CRMP antibody	Prednisone taper slowed to 10 mg q2 weeks; gabapentin 300 mg daily initiated; neuro-oncology referral for outpatient IVIG; PLEX discussed as escalation; infectious/inflammatory labs ordered; PPI + Bactrim continued	20/100
Month 8	6-month follow-up visit neuro-ophthalmology	Color vision OS 4/14; RAPD OS 2+; HVF 24-2: OD MD + 0.24 (non-specific central spots), OS MD −10.24 (cecocentral scotoma + nasal steps); RNFL OCT OD 96 µm, OS 45 µm (significant thinning); GCL OCT OD vol 0.96 mm³, OS vol 0.59 mm³; fundus: resolved disc edema OS, mild temporal disc pallor OS; resolved intraretinal hemorrhages and exudates; melanoma stable	Ongoing monthly IVIG (initiated by neurology); rituximab under consideration for recurrent CSF pleocytosis; melanoma managed by oncology	20/20 (recovered)

AQP-4, aquaporin 4; AMS, altered mental status; CRP, c-reactive protein; CRMP-5, collapsin response mediator protein-5; ED, emergency department; EOM, extraocular movements; FA, fluorescein angiography; GCL, ganglion cell layer; HVF, Humphrey visual field; ICI, immune checkpoint inhibitor; IgG, immunoglobulin G; INL, inner nuclear layer; IPL, inner plexiform layer; irAE, immune related adverse event; IVIG, intravenous immunoglobulin; IVMP, intravenous methylprednisolone; LP, lumbar puncture; MD, mean deviation; MRI, magnetic resonance imaging; MRV, magnetic resonance venography; OCT, optical coherence tomography; OD, right eye; OP, opening pressure; OPL, outer plexiform layer; OS, left eye; PLEX, plasma exchange; PPI, proton pump inhibitor; QFT, Quantiferon tuberculosis; RAPD, relative afferent pupillary defect; RNFL, retinal nerve fiber layer; RPR, rapid plasma reagin.

## 6 Discussion

To our knowledge, this is the first reported case of bilateral neuroretinitis following combination nivolumab and ipilimumab therapy for cutaneous melanoma. Only one prior case of ICI-associated neuroretinitis has been reported in the literature, specifically bilateral neuroretinitis with anterior uveitis following ipilimumab monotherapy as described by Hahn and Pepple in 2016. Neuroretinitis has not previously been described with anti-PD-1 monotherapy or combination checkpoint blockade (4). This is important as the nivolumab-ipilimumab regimen carries the highest systemic irAE burden of any approved ICI regimen, yet remains poorly characterized as a cause of this specific posterior segment complication.

One of the critical diagnostic steps in elucidating the likely etiology of our patient’s neuroretinitis was exclusion of infectious causes, such as *Bartonella henselae*, *Treponema pallidum*, *Mycobacterium tuberculosis*, *Borrelia burgdorferi*, and *Toxoplasma gondii*, all capable of producing a similar clinical presentation and fundoscopic appearance. The patient had no epidemiologic risk factors, and serologies were negative at multiple timepoints. The normal opening pressure, absence of pleocytosis, unremarkable cytology, and negative aquaporin-4 (AQP4)-IgG on CSF analysis provided additional evidence against infectious, neoplastic, or demyelinating etiologies. The absence of optic nerve enhancement on MRI also helped to rule out potential ICI-associated optic neuritis (7).

Interestingly, for our patient, her visual symptoms emerged not during active ICI infusion, but as systemic immunosuppressive therapy was withdrawn. This temporal relationship of ocular inflammation surfacing during steroid tapering has been observed in other ICI-associated posterior segment irAEs and likely reflects steroid-dependent disease rather than a new independent inflammatory event (8). This raises the question of whether active ophthalmologic surveillance should be implemented at the time when a patient develops multisystem irAEs and/or when a patient is placed on steroid therapy for irAEs, rather than initiating ophthalmic examination with onset of symptomatic visual complaints. Whether prophylactic ocular monitoring should become standard practice in ICI-treated patients with multisystem irAEs warrants further investigation and guideline (9).

Our patient received a stepwise treatment approach with acute IVMP pulse and IVIG during initial hospitalization producing partial recovery from 20/400 to 20/200 within days. Subsequent outpatient oral steroid courses led to meaningful but not complete improvement of her visual loss. Therefore, a decision was made to include monthly IVIG therapy outpatient along with her oral steroid taper. This represents a pivotal new step in her treatment as the only previously described ICI-induced neuroretinitis case was treated solely with topical and oral steroids, which still resulted in successful visual recovery in that case (4). IVIG has been used as adjunctive therapy in other corticosteroid-refractory inflammatory optic neuropathy, with case series reporting meaningful recovery and a steroid-sparing effect; therefore, motivating us to pursue such treatment route for our patient (10, 11).

However, despite acuity recovery to 20/20, our patient still has notable functional and structural deficits: reduced color vision and abnormal peripheral vision along with evidence of optic atrophy. This dissociation between Snellen acuity and the broader profile of optic nerve injury is well recognized in inflammatory optic neuropathies and highlights the importance of comprehensive neuro-ophthalmic assessment (such as color vision testing, RAPD evaluation) with ancillary ophthalmic analyses (HVF testing and OCT analysis), rather than relying on chart acuity alone as a marker of recovery in ICI-associated optic nerve disease (12). PLEX was discussed as an escalation strategy should vision deteriorate given published series reported visual improvement in 45–56% of eyes treated with PLEX for severe optic neuritis that were minimally responsive to steroids (13, 14).

As a single case report, we understand the limitation that this study cannot establish a definitive causal relationship between combination nivolumab-ipilimumab therapy and neuroretinitis. Rather, the diagnosis is based on the temporal association with immune checkpoint inhibitor therapy, exclusion of alternative infectious and demyelinating etiologies, negative paraneoplastic antibodies, and the patient’s clinical response to immunosuppressive treatment. Drug rechallenge was neither clinically appropriate nor ethically justified given the severity of the suspected immune-related adverse event, precluding stronger evidence of causality. Although extensive evaluation supported our diagnosis of ICI-associated neuroretinitis, other uncommon inflammatory or paraneoplastic etiologies cannot be excluded with absolute certainty.

In summary, neuroretinitis should be included in the differential for any ICI-treated patient presenting with optic disc edema, intraretinal exudates on OCT scans, and vision loss. Providers should have high clinical suspicion for potential posterior segment irAEs when patient receiving (or who received) combination treatment with nivolumab and ipilimumab presenting with new visual symptoms. Stepwise escalation with IVMP, IVIG, and PLEX can yield meaningful visual recovery. Symptoms may emerge or worsen at the end of steroid tapers for concurrent irAEs, mandating proactive ophthalmologic monitoring. Moreover, sole reliance on visual acuity monitoring is not adequate. These patients with ICI-associated neuro-ophthalmic complications should receive multimodal evaluation to accurately characterize the extent of injury and guide ongoing management. Nonetheless, future research with larger case-series is needed to better characterize ICI neuro-ophthalmic complications, such as neuroretinitis, in hope of creating optimal diagnostic and treatment guidelines to achieve best visual outcome for the patients.

## 7 Patient perspective

The onset of vision loss was sudden and frightening. She recalls waiting too long before seeking care, by which time her vision had already deteriorated significantly. Vision problems had not been on her radar given everything she was already managing with her cancer diagnosis, and the new complication caught her entirely off guard. The vision loss profoundly affected her daily life as she lost the ability to drive and described persistent visual disturbances that required constant attention. She has experienced grief over the loss of vision and continues working toward acceptance.

Treatment has been demanding. She had already been cycling on and off high-dose steroids, and the side effects were frustrating. Monthly IVIG infusions consume approximately two days each month and reliably trigger headaches lasting the remainder of that week, requiring additional management on top of her ongoing recovery.

She emphasizes that any vision change should be evaluated promptly and wishes she had been given clearer information earlier about potential ocular side effects of immunotherapy. Her care ultimately required coordination across multiple specialists, with a neurooncologist serving as a key player in facilitating IVIG approval and guiding her ongoing management. She hopes that greater awareness among both patients and physicians will help others with ICI-associated complications seek care sooner.

## 8 Conclusion

ICI-associated neuroretinitis is rare but vision-threatening and may emerge during corticosteroid tapering for concurrent irAEs. It requires prompt recognition along with aggressive multidisciplinary immunosuppression once infectious etiologies were excluded. Stepwise escalation with IVMP, IVIG, and close follow-up can yield meaningful visual recovery. Comprehensive ophthalmic evaluation and monitoring for ICI-associated neuroretinitis is essential to fully characterize recovery and guide ongoing care.

## Data Availability

The original contributions presented in the study are included in the article/supplementary material. Further inquiries can be directed to the corresponding author.
